# The central role of the centrosome

**DOI:** 10.7554/eLife.84659

**Published:** 2022-12-12

**Authors:** Isabel Stötzel, Eva Kiermaier

**Affiliations:** 1 Life and Medical Sciences (LIMES) Institute, Immune and Tumor Biology, University of Bonn Bonn Germany

**Keywords:** microglia, efferocytosis, centrosome, microtubules, apoptosis, phagocytosis, Zebrafish

## Abstract

The centrosome decides which branch extending from the body of microglia will successfully engulf and clear away dead neurons.

**Related research article** Möller K, Brambach M, Villani A, Gallo E, Gilmour D, Peri F. 2022. A role for the centrosome in regulating the rate of neuronal efferocytosis by microglia in vivo. *eLife* **11**:e82094. doi: 10.7554/eLife.82094.

Cells that are dead or preparing to die through apoptosis must be efficiently removed to maintain healthy tissues during embryo development and also in adults. These cells are detected by specialized immune cells called macrophages, which engulf the unwelcome cellular material via a process termed phagocytosis. Failure to correctly identify and clear cellular waste can result in chronic inflammatory diseases, congenital defects or even cancer (Romero-Molina et al., 2022).

A population of macrophages called microglia are responsible for carrying out this role in the developing brain (Park et al., 2022). However, the mechanism microglia use to efficiently clear dead cells, especially dying neurons, is not fully understood. Now, in eLife, Francesca Peri and colleagues from the University of Zürich – including Katrin Möller as first author – report that a tiny organelle called the centrosome limits the rate at which microglia can engulf and remove cellular debris (Möller et al., 2022).

Most non-dividing cells have a single centrosome, and the cytoskeleton – the network of proteins that gives cells their shape and organizes their internal structures – is made from microtubule filaments that extend from this centrosome (Boveri, 1887; Bornens, 2012; Wong and Stearns, 2003). Using high resolution in vivo imaging, Möller et al. showed that microglia in the brains of zebrafish embryos wipe out dying neurons mainly by extending long branches that embrace and internalize cellular waste. They also demonstrate that this process depends on an intact microtubule cytoskeleton, as destablizing the microtubule filaments using a photoswitchable compound led to changes in cell shape and the loss of cellular extensions (Figure 1). Despite lacking a functional microtubule cytoskeleton and being unable to form cellular branches, the microglia were still able to phagocytose unwanted material but only at their cell body. This suggests that there are several mechanisms by which microglia can phagocytose, ensuring that dead or dying neurons can still be efficiently removed even if some of these processes fail.

**Figure 1.**
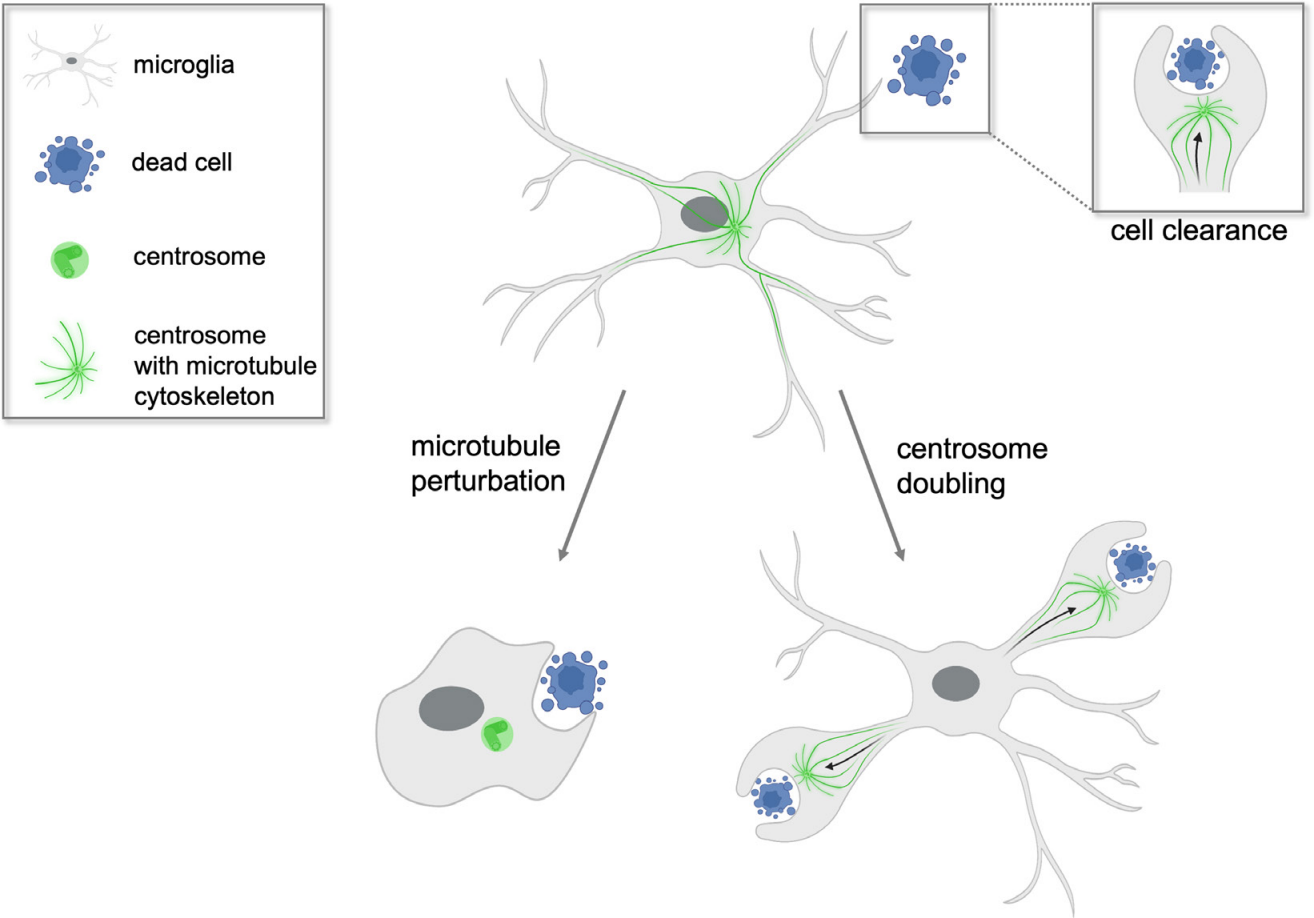
Centrosomes determine which microglial branch will successfully phagocytose cellular waste. Microglia (grey) found in the brains of zebrafish play a crucial role in clearing dead cells (blue) and cellular debris in a process called phagocytosis. Non-dividing microglia have a single centrosome (green) which modifies the cell’s network of microtubules to form branches that can internalize cellular waste. The centrosome then relocates from the cell body to a single branch where successful phagocytosis will occur (top right inset). When the microtubules of the microglia are perturbed experimentally (left arrow), the cell loses its characteristic branched shape and phagocytoses the dead cell at its cell body instead. In contrast, when the centrosome is artificially doubled (right arrow), the microglia is able to engulf dead cell matter in two of its branches simultaneously. This suggests the centrosome plays a central role in determining which branch will be able to execute phagocytosis successfully.

Image credit: Isabel Stötzel, created with BioRender (CC BY 4.0).

Microglia typically only clear one apoptotic neuron at a time even if they are surrounded by several dying cells. Möller et al. therefore sought to investigate the underlying mechanisms that determine the rate of engulfment. They found that the centrosome travelled to the part of the microglia internalizing the unwanted cellular waste, known as the phagosome, just as efficient phagocytosis occurs. The centrosome moves randomly within the cell body during unsuccessful phagocytic attempts that are aborted before engulfment, but relocates from the cell body into single branches when the microglia undergo successful phagocytosis. The team noticed that endosomes, which sort and transport internalized materials into vesicles, also move with the centrosome into the branch where efficient phagocytosis will occur. Thereby the centrosome promotes targeted vesicle transport during phagocytosis.

Based on these results, Möller et al. propose that when the centrosome moves into a particular cellular extension it pre-determines that this branch will be the one that removes the unwanted material. But what happens to phagocytosis when two centrosomes are present in the microglia? To investigate, Möller et al. genetically modified zebrafish to have double the number of microglial centrosomes. The mutant microglia were observed to efficiently engulf apoptotic cells at two cellular extensions simultaneously, with each centrosome relocating to a separate branch (Figure 1). This suggests that the centrosome is the factor that limits the rate at which microglia can clear dead and apoptotic cells, and explains why normal microglia, which have a single centrosome, can only engulf one cell at a time.

Recent findings in macrophages and dendritic cells point to a similar role for the centrosome in improving how the immune system responds to structures that may not belong in the body (Vertii et al., 2016; Weier et al., 2022). In macrophages, the centrosome undergoes maturation upon encountering antigens, whereas dendritic cells increase centrosome numbers under inflammatory conditions. Both scenarios had a positive effect and increased the efficiency of the immune response.

The centrosome has also been shown to reorganize the microtubule cytoskeleton during the formation of the immune synapse, the interface between T cells and antigen-presenting cells. During this interaction, the centrosome moves towards the immune synapse to ensure the delivery and secretion of molecules into the small space between the two cells (Kupfer et al., 1983; Stinchcombe et al., 2006). This guarantees specific killing or T cell activation while minimizing off-target effects. Analogous to what happens in the immune synapse, repositioning of the centrosome and endosome in microglia from the cell body to the forming phagosome correlates with the efficient removal of dead and dying neurons. This suggests a high degree of conservation between the immunological synapse and the phagocytic synapse that connects the microglial cell to the material its internalizing.

Overall, these findings raise several interesting questions. For instance, do the phagocytic and the immunological synapse share other common features, and what is the precise role of the centrosome and microtubule filaments at the phagocytic synapse? In particular, it will be interesting to clarify how centrosomes reorient into one single branch and how they mediate efficient phagocytosis. Future work is also needed to determine the underlying mechanism that allows the centrosome to carry out its role in phagocytosis during development and in adult tissues.
